# A Retrospective Multicenter Analysis of Osteopathic Manipulation in Academic and Community Settings

**DOI:** 10.7759/cureus.83755

**Published:** 2025-05-08

**Authors:** Stephen K Stacey, Bennett Harmelink, Bryan Gordon, Jiwan Toor, Anthony Furlano, Joanne Genewick, Erin Westfall

**Affiliations:** 1 Family Medicine, Mayo Clinic Health System, La Crosse, USA; 2 Family Medicine, Mayo Clinic Health System, Eau Claire, USA; 3 Family Medicine, Mayo Clinic Health System, Mankato, USA

**Keywords:** back pain, chronic pain, health service research, medical coding, musculoskeletal pain, neck pain, resource shortage, retrospective studies, somatic dysfunction, spinal manipulative therapy

## Abstract

Introduction

Osteopathic manipulative treatment (OMT) has a first-line recommendation from the American Academy of Family Physicians and American College of Physicians for the treatment of chronic neck and low back pain, but it remains underutilized. Understanding patterns in the use of OMT in various treatment settings can help guide strategies for improving patient access to this valuable treatment modality.

Methods

We performed a retrospective chart review of diagnosis (International Classification of Diseases, 10th Revision (ICD-10)) and procedure (CPT) codes from a large, multicenter health system during the period of January 2022 through May 2023. We compared the volume of use between academic and community settings. Primary diagnoses and somatic symptom diagnoses were analyzed to determine indications for the use of OMT, and these indications were compared against evidence-based guidelines.

Results

Our search revealed 10,304 outpatient visits where OMT was utilized. OMT addressing the cervical, thoracic, and lumbar spine accounted for 3799 (15.07%), 4193 (16.63%), and 3891 (15.43%) of the total body regions treated during these visits. Visits utilizing OMT most commonly treated three to four body regions (4541; 44.1%), followed by five to six body regions (3004; 29.2%).

Conclusions

Overall, the utilization of OMT remains low across our health system. Further research is needed to determine the best support for current and future physicians to utilize OMT for evidence-based indications.

## Introduction

Osteopathic manipulative treatment (OMT) is an effective first-line treatment for common musculoskeletal complaints such as back and neck pain [1]. Although OMT is recommended as a first-line therapy by the American Academy of Family Physicians and the American College of Physicians to decrease treatment costs and improve pain and function, its use appears to be declining [2-4]. This study aimed to expand current understanding of OMT utilization by assessing its frequency and indications within a large, integrated health system that includes both destination medical centers and community-based practices. Quantifying current indications and utilization rates of OMT in multiple settings provides critical insight to inform targeted organizational strategies aimed at promoting its use as an effective, accessible, and low-cost intervention for pain reduction and functional improvement.

Musculoskeletal symptoms comprise a significant portion of patient complaints, with a rising prevalence over the past 30 years and an estimated 50% increase projected by 2050 [5]. One study estimated that 10-27% of all primary care visits involve musculoskeletal complaints [6]. In fact, approximately 70% of all musculoskeletal complaints are initially treated in primary care settings [7]. While most patients with spinal pain improve with typical strategies such as early mobilization and over-the-counter pain medications, some of these patients go on to develop chronic pain.

Studies on the effectiveness of OMT for chronic back pain have shown varying results but an overall net benefit for specific indications [8,9]. In one randomized controlled trial of 400 participants with chronic low back pain, no significant difference in pain reduction was observed between OMT and a sham treatment at three months, raising questions about the durability of short-term effects in some populations [10]. In contrast, another study reported clinically significant improvement in chronic low back pain at eight weeks, with a number needed to treat of 7 [11]. Furthermore, a systematic review and meta-analysis that included seven studies of 769 participants with chronic low back pain found that both pain and functional status improved after OMT [12]. Taken together, the available evidence supports current guidelines from the American Academy of Family Physicians and the American College of Physicians, which recommend OMT and spinal manipulation as one of several first-line nonpharmacologic therapies for chronic neck pain and low back pain [2,3].

Despite the first-line indication of OMT, most osteopathic physicians do not use it, and its use has been steadily declining over the past several decades [4,8,13]. A survey performed in 1996 revealed that 71% of surveyed physicians used OMT with 5% or more of their patients [14]. Another survey in 1998 revealed that 69.8% of surveyed osteopathic family medicine physicians used OMT with 5% or more of their patients [15]. More recent surveys have shown a further decline in OMT use. A study performed in 2021 revealed only 22.5% of surveyed physicians used OMT with more than 5% of their patients [13], while a 2024 study reported that 43% of DOs nationwide offered OMT, but half of them provided OMT to less than 5% of their patients [16].

By understanding more about the setting (e.g., community vs. academic) and indications for OMT use, healthcare leaders can make more informed decisions on how to direct resources to address this deficit. In this study, we aimed to determine how often OMT was performed and for which indications based on diagnosis (International Classification of Diseases, 10th Revision (ICD-10)) and procedure (CPT) codes in a large, multicenter, multispecialty healthcare system. Although prior data show a decline in OMT use over time, the distribution and frequency of its use remain unclear. Most prior data were collected from surveys, which can lead to distortions from recall bias, social desirability bias, and the availability heuristic. By including actual use data from diagnosis and procedure codes, we hoped to better understand current utilization patterns of OMT and encourage its use as an effective, readily available, low-cost tool to decrease pain and improve function.

## Materials and methods

A retrospective analysis was conducted via record reviews from our hospital system’s electronic health record (Epic). A data analyst exported records of all encounters in which a CPT code for OMT was entered between January 2022 and May 2023 within a large, multicenter healthcare organization serving rural and urban populations in the United States (Mayo Clinic Rochester, Mayo Clinic Jacksonville, Mayo Clinic Phoenix, and Mayo Clinic Health System, a large multispecialty system with locations in MN, WI, and IA). For each of these encounters, we collected the following data: location of the performing provider, all ICD-10 diagnoses for the encounter, and the CPT code entered (98925 for treatment of 1-2 body regions, 98926 for 3-4 body regions, 98927 for 5-6 body regions, 98928 for 7-8 body regions, and 98929 for 9-10 body regions).

We totaled the number of procedures that were performed in two different settings. The first setting included procedures performed at a large, academic medical center or a nearby clinic (defined as sites within Mayo Clinic Rochester, Mayo Clinic Jacksonville, and Mayo Clinic Phoenix), and the second included procedures performed at community medical centers or clinics not in the immediate vicinity of a destination medical center (defined as sites within Mayo Clinic Health System).

The ICD-10 codes were ordered based on the number of times each was used as the primary diagnosis in an encounter. All diagnoses that were used in at least four encounters were separated into one of 13 categories based on body region or treatment category: head, cervical, thoracic, lumbar, sacral, pelvic, lower extremity, upper extremity, chest or rib, abdomen, other musculoskeletal diagnosis, nonspecific pain diagnosis, and general health diagnosis. For example, diagnoses such as "neck pain," "cervicalgia," "cervical somatic dysfunction," and "spinal stenosis, cervical region" were categorized into "cervical diagnosis." The category “other musculoskeletal diagnosis” was used for diagnoses that related to the musculoskeletal system but not a particular body region such as “hypermobility syndrome” and “other specified disorders of bone.” Other pain-related complaints that did not localize to a certain body region such as “fibromyalgia” or “chronic pain syndrome” were categorized into “nonspecific pain diagnosis.” The category “general health diagnosis” included diagnoses such as "essential hypertension," "encounter for immunization," and "encounter for general adult medical examination."

When billing for OMT, treating providers included a somatic dysfunction ICD-10 code for each treated body region (M99.00 for head, M99.01 for cervical, M99.02 thoracic, M99.03 for lumbar, M99.04 for sacral, M99.05 for pelvic, M99.06 for lower extremity, M99.07 for upper extremity, M99.08 for rib, and M99.09 for abdomen). We aggregated the total number of each somatic dysfunction diagnosis entered during any visit where an OMT CPT code was used.

The objective of this study was to provide an overview of real-world practice, rather than to test hypotheses or compare groups. Descriptive statistics were used to characterize the scope and patterns of OMT use. The analysis focused on identifying common clinical indications, anatomical regions treated, and overall procedural volumes.

## Results

**Figure 1:**
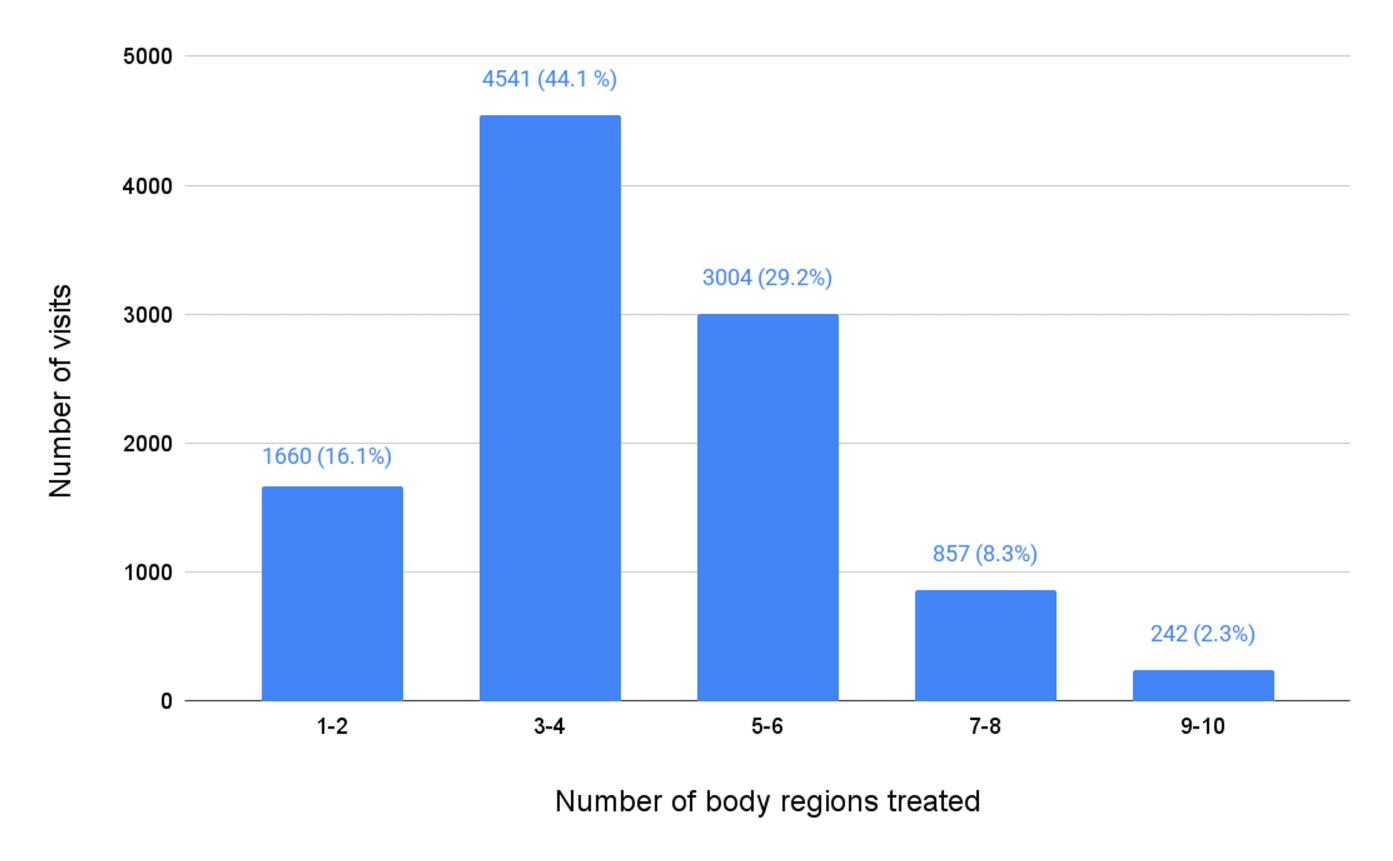
Total number of OMT procedures performed at any Mayo Clinic or Mayo Clinic Health System site between January 2022 and May 2023, by number of body regions treated (n = 10,304). OMT: osteopathic manipulative treatment.

Between January 2022 and May 2023, a total of 10,304 OMT procedures were performed at any Mayo Clinic sites. The majority (88.0%) were performed in community settings (Mayo Clinic Health System Minnesota and Wisconsin), while the remaining (12.0%) were performed at or near a destination medical center (Mayo Clinic Rochester, Mayo Clinic Jacksonville, or Mayo Clinic Phoenix). To contextualize these numbers, Mayo Clinic Health System had 15,000 staff members at the time, compared with a combined total of 61,000 staff members across its sites in Rochester, Jacksonville, and Phoenix [17]. During visits where an OMT procedure was performed, three to four body regions were most commonly treated (4541; 44.1%), followed by five to six body regions (3004; 29.2%) and one to two body regions (1660; 16.1%) (Figure 1).

Each of the 10,304 encounters had a single primary diagnosis coded in the encounters. Of these, 8543 of the primary diagnoses (82.9%) were categorized by body region, having been billed in four or more separate encounters. Cervical diagnoses were the most common (2,116, 24.8%), followed by lumbar spine diagnoses (1,916, 22.4%) and head diagnoses (931, 10.9%) (Table 1).

**Table 1:** Categories of primary diagnoses given during encounters where an OMT was coded. OMT: osteopathic manipulative treatment.

Body region	Number (%)
Cervical spine	2116 (24.8)
Lumbar spine	1916 (22.4)
Head	931 (10.9)
Nonspecific pain	821 (9.6)
General health	617 (7.2)
Thoracic spine	529 (6.2)
Lower extremity	438 (5.1)
Upper extremity	304 (3.6)
Chest or rib	232 (2.7)
Sacral	219 (2.6)
Abdomen	149 (1.7)
Pelvis	143 (1.7)
Other musculoskeletal	128 (1.5)
Total	8543

**Figure 2:**
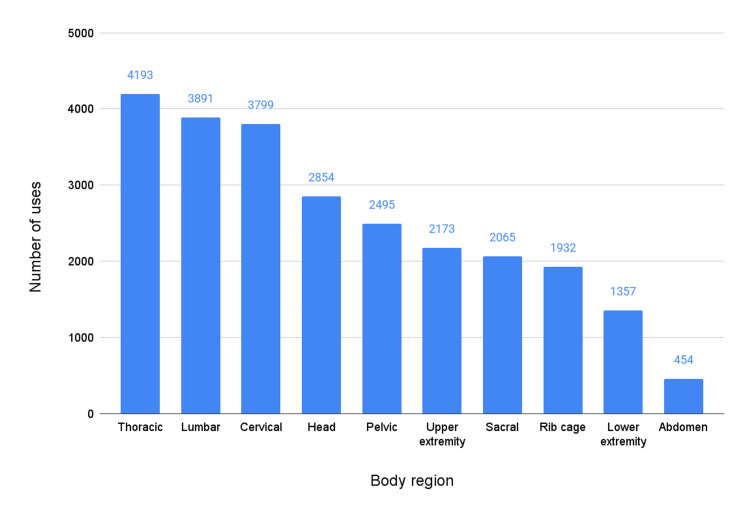
All somatic dysfunction diagnoses (n = 25,213) given during visits in which an OMT procedure was coded at any Mayo Clinic site from January 2022 to May 2023. OMT: osteopathic manipulative treatment.

During these same visits, 25,213 somatic dysfunction diagnoses were coded. Diagnoses of the thoracic region were the most prevalent (4193, 16.6%), followed by lumbar region dysfunction (3891; 15.4%), cervical region dysfunction (3799; 15.1%), and head dysfunction (2854; 11.3%) (Figure 2).

## Discussion

Our data show that within our large, multicenter healthcare system, between January 2022 and May 2023, OMT was most often utilized for thoracic, cervical, and lumbar diagnoses. It was more commonly used in community settings compared to the larger academic centers. OMT was typically applied to three to four body regions, followed by five to six regions. This could indicate several things, including a comprehensive, whole-body approach to the diagnosis of somatic dysfunction. However, the logistics of billing for OMT also encourage treating multiple body regions.

OMT is used at Mayo Clinic and the Mayo Clinic Health System for the most evidence-based indications of chronic neck and low back pain. Lower quality evidence supports the treatment of thoracic [18,19] and head diagnoses [20,21], which were also commonly treated regions. Overall, OMT was utilized in only 10,304 of the 5.2 million outpatient visits (0.20%) during our data collection period [22]. Given the estimated lifetime prevalence of neck pain and back pain is between 20% and 80%, this represents a great opportunity to improve patient outcomes. While there is no published evidence on how often OMT is indicated, the high prevalence of neck and back pain and its recommendation as a first-line nonpharmacologic treatment suggest that OMT is likely underutilized in this healthcare system.

Analyzing the practice patterns of physicians across large health systems who use OMT can help better understand the current utilization rate of OMT and encourage its use as an effective, readily available, low-cost tool to decrease pain and improve function. This approach could address the decline in the use of OMT nationwide, potentially reversing the concerning downward trend by exploring opportunities for physicians to employ this technique effectively. The prevalence of musculoskeletal complaints is increasing, along with the use of long-term medications, such as opioids, and their associated risks [5]. OMT is a supported treatment modality to treat musculoskeletal complaints safely and effectively.

The results of our study align with previous analyses on the prevalent applications of OMT, with spinal indications being the most thoroughly researched and frequently employed [23]. Additionally, consistent with prior studies, we found that overall OMT utilization across the healthcare system was low. However, when it was used, three or more body regions were treated [4]. Our study found a higher use of OMT in community settings compared to destination medical centers. Previous research has not focused on this same comparison, but it does support that OMT is utilized more often in office-based rather than hospital settings [13].

Because primary care physicians are most likely to utilize OMT, our study comparing the use of OMT between community settings and academic settings may be limited by the disproportionately high number of primary care physicians practicing at community sites compared to the large academic institutions [24]. Additionally, while this study has findings from four states in multiple regions of the United States, it only represents a single institution and is susceptible to coding inconsistencies or provider variation in documentation practices. Further research may reveal whether this is a common finding in other systems and regions.

## Conclusions

In this multicenter health system OMT was primarily utilized for cervical and lumbar diagnoses. It was used more frequently in MCHS than in Mayo Clinic sites. Additionally, treatment was most commonly targeted at three to four body regions. The data indicate that physicians are performing OMT in accordance with evidence-based guidelines, although overall utilization appears to be low. Further research aimed at enhancing the ability of osteopathic physicians to employ OMT is essential.
